# SARS-CoV-2 and the Nervous System: From Clinical Features to Molecular Mechanisms

**DOI:** 10.3390/ijms21155475

**Published:** 2020-07-31

**Authors:** Manuela Pennisi, Giuseppe Lanza, Luca Falzone, Francesco Fisicaro, Raffaele Ferri, Rita Bella

**Affiliations:** 1 Department of Biomedical and Biotechnological Sciences, University of Catania, Via Santa Sofia, 97–95123 Catania, Italy; manuela.pennisi@unict.it (M.P.); drfrancescofisicaro@gmail.com (F.F.); 2 Department of Surgery and Medical-Surgical Specialties, University of Catania, Via Santa Sofia, 78–95123 Catania, Italy; 3 Oasi Research Institute–IRCCS, Via Conte Ruggero, 73–94018 Troina, Italy; rferri@oasi.en.it; 4 Epidemiology Unit, IRCCS Istituto Nazionale Tumori “Fondazione G. Pascale”, Via Mariano Semmola, 53 –80131 Naples, Italy; l.falzone@istitutotumori.na.it; 5 Department of Medical and Surgical Sciences and Advanced Technologies, University of Catania, Via Santa Sofia, 87–95123 Catania, Italy; rbella@unict.it

**Keywords:** coronavirus, COVID-19, neurotropism, neuroinvasion, neurovirulence, immune response, brain imaging, cerebrospinal fluid, molecular mechanisms

## Abstract

Increasing evidence suggests that Severe Acute Respiratory Syndrome-coronavirus-2 (SARS-CoV-2) can also invade the central nervous system (CNS). However, findings available on its neurological manifestations and their pathogenic mechanisms have not yet been systematically addressed. A literature search on neurological complications reported in patients with COVID-19 until June 2020 produced a total of 23 studies. Overall, these papers report that patients may exhibit a wide range of neurological manifestations, including encephalopathy, encephalitis, seizures, cerebrovascular events, acute polyneuropathy, headache, hypogeusia, and hyposmia, as well as some non-specific symptoms. Whether these features can be an indirect and unspecific consequence of the pulmonary disease or a generalized inflammatory state on the CNS remains to be determined; also, they may rather reflect direct SARS-CoV-2-related neuronal damage. Hematogenous versus transsynaptic propagation, the role of the angiotensin II converting enzyme receptor-2, the spread across the blood-brain barrier, the impact of the hyperimmune response (the so-called “cytokine storm”), and the possibility of virus persistence within some CNS resident cells are still debated. The different levels and severity of neurotropism and neurovirulence in patients with COVID-19 might be explained by a combination of viral and host factors and by their interaction.

## 1. Introduction

### 1.1. General Epidemiological and Clinical Features

Coronaviruses (CoVs) are a group of large enveloped non-segmented positive-sense RNA viruses, causing respiratory and enteric diseases in animals and humans [1]. A highly pathogenic CoV, named Severe Acute Respiratory Syndrome (SARS)-CoV-2 (formerly known as 2019-nCoV), emerged in December 2019 in the Hubei region of China, and in the city of Wuhan in particular. The initial cases, presenting with a dry cough, sore throat, fever, dyspnea, and bilateral lung infiltrates on chest imaging, were all linked to the Wuhan’s Huanan Seafood Wholesale Market, which trades fish and a variety of live animals, including bats, poultry, marmots, and snakes [2]. This novel CoV has caused an outbreak of severe pneumonia (called COVID-19) in China, that has rapidly spread around the world [3]. On 30 January 2020, the World Health Organization (WHO) declared the epidemic an international public health emergency and, a few weeks later, a global pandemic [4].

Person-to-person transmission occurs primarily through direct contact or droplets spread by coughing or sneezing from infected individuals [5]. In closed rooms, larger aerosols with a greater contagion capacity can form, in which the virus lasts for several hours. Transmission by fomites is also possible as SARS-CoV-2 persists on smooth surfaces (such as cardboard, stainless steel, and plastic surfaces) from 24 to 72 h. Additionally, SARS-CoV-2 has been detected not only in lung secretions but also in blood, feces, saliva, and urine of infected people [6].

The symptoms of COVID-19 infection usually appear after an incubation period of approximately 5 days (from 3 to 14 days) [7] and, in deceased patients, the interval from the symptom onset to death ranges from 6 to 41 days, with a median of 14 days [8]. Most SARS-CoV-2 infected patients have mild symptoms that spontaneously resolve, whereas others, especially comorbid adults and elderly subjects, develop various complications, including severe pneumonia, septic shock, pulmonary edema, and acute respiratory distress syndrome, despite invasive ventilation in some cases. The overall mortality is estimated to be 8% and is due to respiratory failure or multiple organ failure [9,10].

### 1.2. Basic SARS-CoV-2 Virology

The term “coronavirus” refers to the peculiar crown-shaped appearance of its envelope at electron microscopy, further surrounded by spike-shaped membrane glycoproteins. In particular, the glycoprotein S, located on the outer surface, forms a three-dimensional structure in the receptor-binding domain of the host cell that facilitates the viral anchorage [11]. Phylogenetically, the *Coronaviridae* family encompasses four genera, i.e., alpha-, beta-, gamma-, and delta-coronavirus. SARS-CoV-2 is a beta-coronavirus with a 29,903-base single-stranded RNA genome that belongs to the *Orthocoronavirinae* subfamily, which is part of the *Coronaviridae* family [12].

CoVs have remarkable genetic diversity and a high ability to recombine, thus explaining the interspecies leap of some CoVs that have affected humans in recent decades [13]. The genome of SARS-CoV-2 is closely related to two beta-coronaviruses isolated in bats, and phylogenetic studies suggest that bats are the original host and reservoir [14,15]. Then, it is likely that SARS-CoV-2 jumped into humans through an intermediate host, probably the pangolin [16]. Sequence analysis of SARS-CoV-2 has shown the typical structure of other CoVs [3], in particular, it shares highly homolog sequences with the SARS-CoV [17] and clinically causes pneumonia similar to that previously induced by the SARS-CoV and the Middle East Respiratory Syndrome (MERS)-CoV [18].

Structurally, SARS-CoV has a well-defined composition comprising of 14 binding residues that directly interact with the human angiotensin-converting enzyme-2 (ACE2) receptor. Given the similarity between SARS-CoV and SARS-CoV-2, the pathogenesis underlying these infections may be the same [19,20,21], although the exact pathogenic mechanism of SARS-CoV-2 is not yet completely understood.

### 1.3. Diagnostic Findings and Treatment Possibilities

The WHO issued guidelines for the diagnosis and management of COVID-19 [22]. Overall, its diagnostic criteria take into account the clinical features and the epidemiological risk. Clinical diagnosis is based on a history of exposure and clinical manifestations, supported by laboratory confirmation. The antibody response to SARS-CoV-2 follows a typical pattern, with the IgM antibodies disappearing 12 weeks after initial infection and the IgG antibodies (specifically directed against viral protein S and N) persisting for a longer time, thus likely exerting a protective role [23].

The real-time reverse polymerase-transcriptase chain reaction (RT-PCR) technique and genomic sequencing techniques are valid tests currently used to confirm the diagnosis from the nasopharynx and respiratory secretions. Conversely, the isolation and cultivation of the virus in the blood and the sequencing of its entire genome are limited in the clinical practice due to the high costs involved [24]. Viral antigen and more rapid antibody detection systems (such as the enzyme-linked immunosorbent assay) are currently being developed, although their accuracy is still limited by the relatively high rate of false-negative cases and, therefore, needs to be improved [25]. More recently, other studies are trying to propose droplet digital PCR-based methods as more effective diagnostic strategies for the identification of SARS-CoV-2 positive patients with low viral load [26].

To date, there is no effective treatment for patients with COVID-19. Adenosine analogs (e.g., remdesivir, favipiravir, ribavirin, and galidesivir) acting on the RNA-dependent polymerase and blocking the viral RNA synthesis are promising. Chloroquine (CQ) and hydroxychloroquine (HCQ) can effectively inhibit SARS-CoV-2 in vitro, but their efficacy in vivo is under evaluation, as well as the effect of serum rich in anti-SARS-CoV-2 antibodies obtained from convalescent subjects [23]. Very recently, a systematic review assessed the efficacy and safety of CQ/HCQ for treatment or prophylaxis of adult patients with COVID-19 [27]. Thirty-two studies were included, of which 6 randomized clinical trials (RCTs) and 26 non-randomized, with a total of 29,192 participants. Overall, studies suggest that the treatment of hospitalized patients with CQ/HCQ may not reduce the risk of death compared to standard care. High dose regimens or combination with macrolides may be associated with harm, particularly QTc prolongation and cardiac arrhythmias [28]. Post-exposure prophylaxis may not reduce the rate of infection, although the quality of the evidence is low. The authors concluded that patients should be treated with CQ/HCQ only if monitored and in the context of high-quality RCTs [27]. Therefore, rationalization of the use of these drugs is also advised [29].

Other non-specific immune modulators include human immunoglobulin and corticosteroids, such as dexamethasone, a glucocorticoid that has proved to be the first life-saving drug in these patients. In particular, dexamethasone 6 mg once daily (either per os or by intravenous injection) for 10 days may result in a reduction in mortality by 1/3 in patients on ventilators and by 1/5 in those receiving oxygen [30]. Other treatment options include specific monoclonal antibodies that bind the receptor-receptor domain of SARS-CoV-2 and antibodies that block inflammatory interleukins (IL), such as tocilizumab. Finally, several vaccines are under analysis and include live attenuated viruses, inactivated viruses, use of recombinant DNA, and vaccines based on SARS-CoV-2 specific proteins and subunits [31]. Until these therapeutic options are confirmed, the main measures are prevention, isolation, social distancing, frequent hand washing, and the use of personal protective equipment.

## 2. Background on SARS-CoV-2 and Nervous System

Some experimental and clinical studies previously performed on other CoVs and preclinical models seem to converge on the evidence that these viruses may have a tropism into the central nervous system (CNS). Seven types of CoVs are currently known that can infect humans and cause neurological damage [23]. In some animal and human CoVs (including those causing SARS and MERS), a neuroinvasive potential has been demonstrated [32]. Although there are limitations in the epidemiological studies carried on COVID-19, as well as limited case records for determining the actual incidence of these complications, some patients reported neurological symptoms, but clinical findings and pathogenic features have not yet systematically addressed.

The penetration of several respiratory viruses into the CNS has already been shown, a mechanism called “neuroinvasion,” affecting both glia and neurons [31]. In some cases and under certain conditions, the neurotropism can cause neurovirulence, which refers to the development of neurological manifestations [33]. In the case of SARS-CoV-2, the existence of these phenomena is supported by previous evidence showing human CNS infection from other respiratory viruses, the CNS of other species infected by CoVs, animal and in vitro models of CNS infection by human CoVs, and the occurrence of neurological complications in the course of other human CoVs infections.

The aims of this review are i) to summarize the available information on the relationship between CoVs and the nervous system, ii) to identify the potential targets and routes of entry of SARS-CoV-2 into the nervous system, and iii) to describe the range of the neurological features reported to date in patients with COVID-19 and the proposed pathogenic mechanisms.

### 2.1. CNS Infection From Respiratory Viruses

All the common respiratory viruses affecting humans, such as influenza, CoVs, and respiratory syncytial virus (RSV), can be associated with various neurological manifestations, particularly in subjects experiencing severe pulmonary symptoms [34]. For instance, the effects that RSV may cause include seizures, encephalitis, ataxia, and cerebellitis, and the virus has been detected in the cerebrospinal fluid (CSF). Influenza is known to cause neurological complications, such as encephalitis, myelitis, meningitis, and Guillain-Barré syndrome (GBS) [34].

### 2.2. Coronaviruses Affecting the CNS of Other Species

Respiratory viruses, including the CoV family, affect the CNS of other species, such as birds, felines, and livestock [35]. Meningitis and spinal cord inflammation have been reported in cats affected by a pathogenic feline CoV [36]. A 91% homology resemblance has been assessed between the human OC43 CoV and the swine hemagglutinating encephalomyelitis virus (HEV), which can invade the porcine brain by retrograde neuronal propagation through the peripheral nerves [37]. A subspecies of murine CoVs, called mouse hepatitis virus, induces a demyelinating disease resembling Multiple Sclerosis (MS) [35].

### 2.3. Animal and In Vitro Models of CNS Infection by Human Coronaviruses

Human CoVs can induce acute or persistent infections in neuronal cell lineages, neuroglia, and oligodendrocytes [38,39,40]. Moreover, flaccid paralysis and demyelination in animal models can be caused by the human OC43 CoV [41]. In particular, it has been shown that the spread of the OC43 in susceptible mice runs from the olfactory bulb to the brainstem and spinal cord, and uses the axonal transport system as the avenue for the neuron-to-neuron spread [33]. Further, neuron-to-neuron propagation strategies observed in cell cultures include both passive viral particle diffusion and axonal transport [42]. SARS-CoV, which may enter the CNS through the olfactory bulb and transneuronally spread to other brain regions, can cause neuronal death in the human ACE2 receptor transgenic mouse [43].

### 2.4. Neurological Complications by Other Human Coronaviruses

At least four types of human CoVs have shown neuroinvasive capacity based on the detection of viral RNA or other nucleic acids in the human brain [35].

In a 12-month-old infant with severe immunodeficiency, a case of fatal OC43 CoV-related encephalitis was confirmed through RNA sequencing techniques and RT-PCR in samples of brain biopsy [44]. Immunohistochemical study of the brain showed a microglial reaction, T lymphocyte infiltrates, and presence of the OC43 CoV nucleocapsid in neurons. In a 15-year-old adolescent with disseminated acute encephalomyelitis associated with OC43 CoV infection, magnetic resonance imaging (MRI) disclosed demyelination in the subcortical white matter, cerebellum, and spinal cord [45]. The OC43 CoV was detected in the CSF and nasopharynx secretions by using the PCR. There has also been a report of GBS associated with 229E and OC43 CoV co-infection in a pediatric patient [46].

Encephalitis, ischemic stroke, and polyneuropathy can result from SARS-CoV exposure, with viral RNA detectable in the CSF [47,48]. In a necropsy study carried out on 8 victims of SARS-CoV, infected neurons were found in the cortex and hypothalamus, and genomic sequences of SARS-CoV were detected in all cases by using the RT-PCR [49].

Encephalomyelitis and vasculitis may result also from MERS-CoV infection. A series of three patients showed that they all suffered from an altered level of consciousness, ranging from confusion to coma, along with ataxia and motor deficit [50]. At brain MRI, bilateral lesions were evident in the white matter of the frontal, parietal, and temporal lobes, as well as in the basal ganglia and corpus callosum. Two of these patients showed an increased protein level in the CSF, while all had lymphocytopenia and severe multiple organ involvement, including kidney, liver, and the cardiovascular system [50]. During the MERS-CoV infection, other neurological complications were reported: brainstem encephalitis, GBS [51], and cerebral hemorrhage in the context of thrombocytopenia and disseminated intravascular coagulation [52]. A retrospective study involving 70 MERS patients reported that 8.6% had seizures, while four had GBS in a series of 23 cases. The latency of the neurological symptoms ranged from 7 and 26 days after the onset of the pulmonary disease [53].

## 3. Search Strategy and Results

A PubMed-based literature search was performed to find all relevant reports published until June 2020. The search queries were “COVID-19 AND nervous system”, “brain”, “neurology”, “neurological”, “encephalopathy”, “encephalitis”, “stroke”, “seizures”, “neuropathy”. The search was also repeated by using the above-mentioned keywords and the term “SARS-CoV-2” instead of “COVID-19”.

The authors selected all the articles based on the abstract and full-text examination and without a priori appraisal of inclusion/exclusion criteria. Indeed, the number of studies reporting neurological complications of COVID-19 is still limited and the majority include case reports/case series or retrospective samples, without a systematic specific assessment of the neurological complications. The references of the articles retrieved were also examined in search of more data. After this process, a total of 23 studies [54,55,56,57,58,59,60,61,62,63,64,65,66,67,68,69,70,71,72,73,74,75,76] were included.

**Figure 1:**
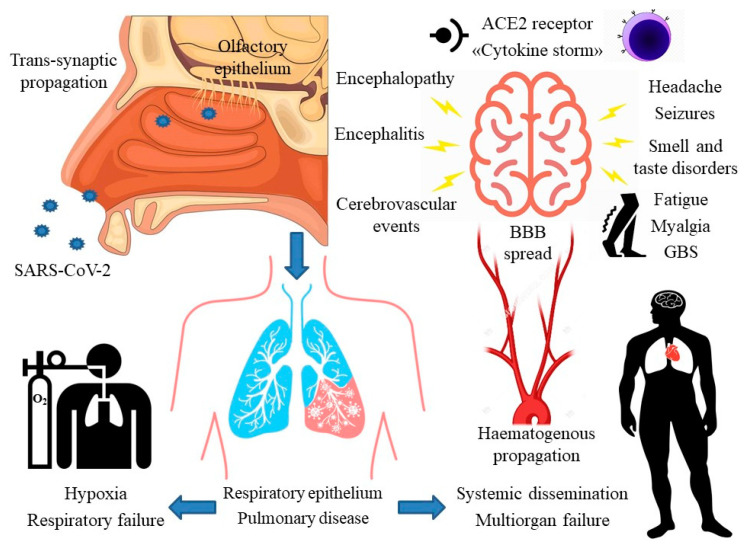
Main neurological manifestations of COVID-19 and proposed mechanisms of SARS-CoV-2 neuroinvasion. ACE2: angiotensin II converting enzyme receptor-2; BBB: blood-brain barrier; GBS: Guillain-Barré syndrome.

Figure 1 summarizes the main neurological manifestations of COVID-19 and proposed mechanisms.

## 4. Main Neurological Manifestations of COVID-19

The available research on the neurological involvement in SARS-CoV-2 makes it hard to causally link a specific neurological manifestation to the viral infection. As a general rule, severe forms of COVID-19 are more likely to produce neurological complications when compared to the mild forms (45.5% versus 30%). An autopsy study on deceased patients with COVID-19 due to respiratory failure indicated the presence of cerebral edema and neuronal degeneration in these subjects [77].

A study in Wuhan (China), reported neurological findings in 214 hospitalized patients with COVID-19 [68]. Another systematic study in France [60] noted neurological symptoms in 49 of 58 patients, including confusion, encephalopathy, and cortico-spinal tract signs at clinical examination, along with leptomeningeal enhancement and perfusion abnormalities on brain MRI. Overall, the most common neurological symptoms reported in some patients with COVID-19 were headache, anosmia, ageusia, asthenia, and myalgia, followed by encephalopathy, seizures, stroke, and encephalitis [78].

### 4.1. Encephalopathy

Elderly patients and those with previous cognitive decline, multiple comorbidities, other infections, severe medical illness, poor premorbid functional status, malnutrition, and vascular risk factors (especially hypertension) have a higher risk to show an altered level of consciousness related to COVID-19 [55,68,79]. Moreover, metabolic or endocrine derangements, including hypo- or hypernatremia, hypo- or hypercalcemia, hypo- or hyperglycemia, renal and/or liver dysfunction, among others, put patients at further risk for encephalopathy. Sepsis and the subsequent inflammatory and the so-called “cytokine storm” may also contribute to encephalopathy with IL-6, IL-8, IL-10, and tumor necrosis factor α (TNFα) being implicated in confusional states [80]. Finally, patients with previous neurological disorders and acute respiratory symptoms seem to be at increased risk for encephalopathy as the initial symptom of COVID-19. Coherently, in the study by Mao et al. [68], 15% of patients with a severe form of the disease presented an altered level of consciousness.

Toxic and metabolic causes, as well as the effects of drugs or hypoxia, may result in COVID-19-associated encephalopathy [57]. Interestingly, an electroencephalography (EEG) report on a patient with altered mental status who was unable to follow verbal orders as the presenting symptom of COVID-19 showed diffuse slow waves, particularly in the left temporal region, whereas pathological findings demonstrated cerebral edema without inflammatory signs [55]. In these cases, treatment is symptomatic and includes fever control, treatment of hypoxia, and antiepileptic medications [77].

A case of COVID-19-associated (confirmed by RT-PCR in a nasopharyngeal sample) acute hemorrhagic necrotizing encephalopathy has also been described [71]. Brain computed tomography (CT) detected a symmetrical bilateral hypodense area in the medial thalamic nucleus, whereas MRI showed contrast-enhanced hemorrhagic lesions, with multifocal and symmetrical disposition, in both thalami, insula, and the mesial region of temporal lobes [71]. Although relatively rare, acute necrotizing encephalopathy can be a severe complication of some viral infections, including the influenza virus. The authors postulated that the pathogenesis might be related to the “cytokine storm” induced by COVID-19 [81]. A posterior reversible encephalopathy-like syndrome, associated with transient cortical blindness, was also reported [64].

### 4.2. Encephalitis

Based on the available evidence, SARS-CoV-2 should be included in the differential diagnosis algorithm of viral encephalitis. Typical symptoms are fever, headache, seizures, behavioral disorders, and altered level of consciousness. In these patients, an early diagnosis is of crucial importance to increase the survival rate, especially in those with severe pneumonia and hypoxia [23]. In a report of a 56-year-old woman from Wuhan with COVID-19, the brain CT remained normal but the diagnosis of encephalitis was confirmed through the isolation of SARS-CoV-2 in the CSF using genomic sequencing techniques [75].

The case of a 24-year-old Japanese man presenting with multiple generalized epileptic seizures and decreased level of consciousness led to a diagnosis of meningoencephalitis [69]. Brain MRI showed hyperintense areas in the right mesial region of the temporal lobe and hippocampus. While the SARS-CoV-2 RNA was not detected in the nasopharynx, it was identified in the CSF by using RT-PCR, although it was unclear if some of the patient features were present in the context of seizures due to other causes [69]. Anyhow, high levels of proinflammatory cytokines in the CSF can cause breakdown and increased permeability of the blood-brain barrier (BBB) which may, in turn, lead to viral invasion and clinical manifestation [71].

### 4.3. Seizures

Seizures have already been reported in CoV infections, and there has been a high proportion of breakthrough seizures in patients with epilepsy who developed COVID-19 [78]. Nevertheless, a recent analysis of 304 patients with COVID-19 [67] reported two seizure-like events only, with no confirmed cases of new-onset seizures. However, the study was limited by a lack of instrumental investigation (e.g., EEG, neuroimaging) and by its retrospective approach.

A case report of a patient with no history of epilepsy who had multiple apparent tonic-clonic seizures in the context of COVID-19 might be interpreted as an unmasked seizure disorder or a direct effect of COVID-19 on the brain, although further confirmations are needed [63].

### 4.4. Cerebrovascular Events

When compared to younger subjects without comorbidities, elderly patients with COVID-19 and vascular risk factors appear to be at greater risk for developing cerebrovascular complications [23]. In a retrospective study of 221 patients [66], 11 (5%) presented with ischemic stroke, one (0.5%) with cerebral venous thrombosis, and one (0.5%) with a cerebral hemorrhage.

The risk factors for stroke in this population were: advanced age (mean age: 71.6 years), severe pulmonary disease, hypertension, diabetes, marked inflammatory or procoagulant response (e.g., increased C-reactive protein and D-dimer), and previous cerebrovascular events [66]. Other researchers described five patients with stroke (80% ischemic), who had severe forms of COVID-19, increased D-dimer, thrombocytopenia, and multiple organ failure [68]. Notably, a study in the USA demonstrated that young patients (aged < 50 years) more likely developed large-vessel strokes in the context of COVID-19, suggesting that all ages are vulnerable [70].

Regarding pathomechanisms, the increased risk of cerebrovascular disease during the COVID-19 infection is likely multifactorial. It has been shown that SARS-CoV-2 can bind to the ACE2 receptor on endothelial cells, which might result in increased blood pressure. Both ischemic and hemorrhagic strokes can be secondary to the increase in blood pressure, together with the presence of thrombocytopenia and coagulation disorders.

The “cytokine storm” may act as another pathogenic mechanism [23]. The levels of C-reactive protein, ferritin, D-dimer, lactate dehydrogenase, and the leukocyte count have often been found to be elevated in these patients [82]. Moreover, increased inflammatory markers and hypercoagulability state seem to characterize severe cases, along with a substantially enhanced risk of stroke [68]. The likelihood of ischemic or hemorrhagic stroke may be also increased by some viral-related mechanisms, including vascular endothelial cell infection and consequent vessel damage. On the other hand, it is well known that infection-associated systemic inflammation, thrombosis, or vasculitis increase the risk of stroke [83]. Finally, systemic vasculitis and CNS vasculitis have been demonstrated at autopsy in patients with SARS-CoV [84].

### 4.5. Headache

Most patients with COVID-19 complain of headaches. Guan et al. [57] found that 13.6% of a series of more than 1000 patients reported headache, and in 15% of those with severe forms. The intensity of headache was generally referred to be mild, although the study did not mention whether a prior history of headache or any meningeal sign was present. In a recent case series [58], headaches were a predominant complaint, along with fever, cough, sore throat, and breathlessness. The prevalence varies in different reports, but headache may affect up to 1/3 of patients [54,62].

Headache is a well-known clinical feature of meningitis, encephalitis, intracranial hypertension, cerebrovascular diseases, and vasculitis, whereas scarce pathophysiological data link it to COVID-19. In some cases, cytokines and chemokines released by macrophages may activate nociceptive sensory neurons [85], with a neuroinflammatory mechanism similar to that involved in pain [86]. In this scenario, screening patients for secondary causes of headache, including COVID-19, is mandatory, especially for patients in whom frequency or severity of headache change, or present with systemic symptoms, or do not respond to first-line or habitual treatments.

### 4.6. Smell and Taste Disorders

Anosmia and secondarily taste disorders are commonly reported in patients with COVID-19 and may appear suddenly [56]. In Italy, 19.4% of patients had some form of chemosensory dysfunction [74], whereas in a case register of twelve European hospitals, the prevalence of olfactory and gustatory dysfunction in 417 patients with COVID-19 with mild-to-moderate symptoms [65] was 85.6% and 88%, respectively. Notably, 12% of them declared the olfactory dysfunction as the initial symptom and 18% had no runny nose or nasal obstruction [65]. Indeed, although anosmia is noted in many other respiratory infections, such as cold and influenza, in COVID-19 it is typically not accompanied by nasal swelling or rhinitis [65,74,87].

Given the reports of anosmia presenting as an early symptom of COVID-19, dedicated testing may offer the potential for early detection of SARS-CoV-2 infection. Nevertheless, the chemosensory deficit in COVID-19 has not yet been systematically investigated, although it is a current research “hot topic”, both at the clinical-epidemiological and cellular level. Initial observations and early studies suggested as possible mechanisms for anosmia in COVID-19 the cleft syndrome, nasal obstruction and rhinorrhea, “cytokine storm”, direct damage to olfactory receptor neurons (ORNs), and impairment of the brain olfactory centers. The most obvious cause would be a direct damage to ORNs, since other human CoVs (e.g., OC43) have shown to directly bind to ORNs. However, although anosmia is linked with human viruses causing the common cold, such as influenza and other CoVs (respiratory or not), the exact mechanism has not yet been clearly established.

A new model of olfactory dysfunction in COVID-19 has been recently drawn from the observation that sustentacular cells (SUSs) are the primary target of the virus and that SUSs infection triggers a cascade of events leading to anosmia [88]. SUSs express ACE2 and would be infected first. Impairment of SUS would negatively affect ORNs, leading to the inhibition of the odor perception. Simultaneously, rapid immune response would be induced in a subset of ORNs and microvillar cells, which would lead to activation of lymphocytes and macrophages and their infiltration into the olfactory epithelium, as well as secretion of proinflammatory cytokines. Stem cell infection may potentially explain why a small fraction of patients with COVID-19 experience long-term dysosmia [88].

Of note, such a model does not imply that SARS-CoV-2 travels from the olfactory epithelium to the brain along the olfactory axons. Indeed, no axonal transport of SARS-CoV-2 to the brain has been demonstrated in the hamster model during the first two weeks after infection [89], and no viral accumulation or persistence has been reported in cerebral olfactory regions of autopsy material from patients with COVID-19 [90]. On the other hand, rapid SARS-CoV-2 accumulation in the brain after intranasal injection was recently shown using the new humanized ACE2 knock-in mouse [91]. Yet, this is not synonymous with transport along olfactory axons, as other routes are also possible. If SARS-CoV-2 travels within the olfactory axons, this would require an ACE2-independent passage of the virus from SUSs to ORNs within the olfactory epithelium. In addition, it would be relevant to examine progenitor or stem cell infection, as these olfactory epithelium cells also express significant levels of ACE2. Probably, also host genetic factors play a role in individual susceptibility to anosmia in COVID-19 and the characterization of these factors is of particular interest [92].

Regarding gustatory dysfunction in COVID-19, it is known that the ability to separate flavors depends on the retronasal stimulation pathway. Therefore, in patients with ageusia, retronasal olfactory dysfunction is commonly suggested, although some studies reported high ACE2 expression on the oral cavity mucosa and epithelial cells of the tongue [93]. Another possibility is that SARS-CoV-2 may have a direct effect on the taste buds or receptors [94].

### 4.7. Guillain-Barre Syndrome

GBS (i.e., acute inflammatory demyelinating polyneuropathy) can follow a gastrointestinal or respiratory infection. A molecular mimicry mechanism, in which infecting viruses likely share epitopes similar to some peripheral nerve components, is believed to occur and to stimulate autoreactive T or B lymphocytes. Antibodies against the virus cross-react and bind to peripheral nerve components, thus causing neuronal dysfunction and clinical manifestations. After SARS- and MERS-CoV infections, both GBS and acute motor axonal neuropathy have been described [32,51].

Reports of GBS in patients with COVID-19 are emerging. A case series [73] reported five cases of GBS in Italy, and four of them presented with lower-extremity weakness and paresthesia. Patients developed symptoms five to 10 days after the onset of the viral infection. At electromyography, two patients had GBS and three acute motor axonal neuropathy [73]. Another patient in Iran [72] and an Italian patient with the Miller-Fisher GBS variant [59] were also reported.

In a 62-year-old patient presenting with motor weakness of the lower extremities and COVID-19 symptoms, GBS associated with SARS-CoV-2 infection was observed a week later [76]. An increase in proteins (124 mg/dl) but not in cells was found in the CSF, while at neurophysiological examination increased distal latencies and F-waves absence was detected, suggesting a severe and diffuse peripheral nerve demyelination. Although the authors reported that the patient was infected by SARS-CoV-2 at the onset of GBS symptoms (because the patient had lymphopenia and thrombocytopenia), it cannot be excluded that COVID-19 and GBS presented coincidentally [76], and therefore further evidence is needed.

### 4.8. Non-Specific Neurological Symptoms

Some of the most frequently described non-specific symptoms are myalgia, unsteadiness, and fatigue. Of note, 36.4% of 214 patients with COVID-19 admitted in a Wuhan hospital exhibited neurological manifestations [68], which were categorized as “CNS involvement” (24.8%), “peripheral involvement” (10.7%), and “muscular-skeletal involvement” (10.7%). Among the latter, 15% of the non-severe patients reported myalgia, while 13.7% had elevated levels of creatine kinase. Two cases of rhabdomyolysis (0.2%) were also described [61].

### 4.9. Neuropsychiatric Manifestations

Apart from a general unspecific reaction to the awareness of infection, some psychiatric illnesses might directly result from exposure to human CoVs. In patients with psychotic symptoms compared to non-psychiatric controls, a higher prevalence of immune reactivity for HKU1 and NL63 CoVs was found [95], suggesting that viral exposure may represent a comorbid risk factor in neuropsychiatric disease. However, the role that SARS-CoV-2 may play in the etiopathogenesis of psychiatric diseases needs to be explored.

## 5. Proposed Neuropathogenic Mechanisms

### 5.1. Lessons from SARS- and MERS-CoV Infections

SARS-CoV entry into the human host cell seems to be mediated primarily by cellular receptors ACE2, which are expressed in the lung, kidney, vascular endothelia, small intestine, and human airway epithelia [96,97,98]. Conversely, MERS-CoV may enter human host cells primarily through the dipeptidyl peptidase-4 (DPP4) protein located in the membrane of cells of the immune system, liver, small intestine, and lower respiratory tract [99,100]. However, ACE2 or DPP4 alone are not enough to make the host cell susceptible to infections. This is particularly true when considering that SARS or MERS infections were also reported in the CNS, where ACE2 or DDP4 expression level is low under normal conditions [101].

The exact route through which SARS and MERS CoVs enter the CNS is still not clear, although the glymphatic or a pure hematogenous path seems to be unlikely, particularly in the initial infection stage, during which no virus particle is detected in the brain [49,102,103]. However, some evidence indicates that CoVs might initially invade peripheral nerve terminals, and later the CNS through a synapse-connected route [104,105,106,107]. Coherently, the SARS infection seems to be able to cause significant neuronal damage without substantial inflammatory infiltration [43].

Earlier studies have shown the presence of viral particles in the brain of patients with SARS, located exclusively in the neurons [102,103]. In vivo experiments using transgenic mice showed that, when SARS or MERS CoVs are given intranasally, they can enter the brain via the olfactory nerve, and quickly spread to specific brain regions, such as the brainstem and the thalamus [43,108]. In these cases, the brain expresses ACE2 receptors, that have been detected in neurons and glial cells, making them a potential target for COVID-19.

Another important observation is that in mice infected with MERS-CoV with low inoculum doses, virus particles are not detected in the lung but only in the brain, which indicates that the CNS infection is relevant for the high mortality of the disease [108]. However, although murine models develop CNS infection, MERS-CoV has never been detected in the human CNS, thus suggesting a different disease model [109].

### 5.2. Transsynaptic Propagation

Viruses are present in the brain of patients with SARS-CoVs [49]. Baig et al. [24] have recently suggested a putative transcribral SARS-CoV-2 route to the brain and the presence of its RNA in the CSF would be the conclusive evidence to support the COVID-19 neurovirulence. However, the pathomechanisms underlying the CNS invasion seem to be more complex.

A CNS invasion can occur in both the initial and late phases of SARS-CoV-2 infection [24]. However, research is yet to determine the exact route for the entrance of the virus into the brain. A direct entry along the olfactory nerve can be considered among the potential mechanisms. In particular, the nasal olfactory epithelium is the probable site of enhanced binding of SARS-CoV-2. Multiple non-neuronal cell types within the olfactory epithelium express two host receptors, ACE2 and transmembrane protease serine 2 (TMPRSS2), that facilitate SARS-CoV-2 binding, replication, and accumulation. Moreover, a subsequent brain infection beginning from the olfactory neurons might be considered, as well as the possibility that ORNs may initiate a rapid immune response at the early stages of the disease [110]. Using a mouse model, Bilinska et al. [111] determined whether cells in the olfactory epithelium expressed the receptors allowing the entry of the SARS-CoV-2 virus. They showed that ACE2 and TMPRSS2 were expressed in the SUSs of the olfactory epithelium but not, or much less, in most olfactory receptor neurons, suggesting that SUSs are involved in SARS-CoV-2 virus entry and smell impairment. Moreover, the expression of the entry proteins increased in older animals, thus possibly explaining, if verified also in humans, why older individuals are more susceptible to SARS-CoV-2 infection [111].

Translationally, these preliminary findings suggest that damage to the olfactory epithelium may not only underlie clinical anosmia but also represent a preferential gate to the brain. Namely, SARS-CoV-2 might spread via the transcribral route from the olfactory epithelium along the olfactory nerve to the olfactory bulb within the CNS or spread retrogradely via transsynaptic transfer using an endocytosis or exocytosis mechanism and a fast axonal transport mechanism of vesicle transport moving the virus along microtubules back to neuronal cell bodies [78]. Additionally, another possible transsynaptic route from the nasal respiratory epithelium to the brain via the trigeminal nerve branch has recently been hypothesized, although replication of the findings is needed [112].

The biological plausibility of the retrograde transsynaptic pathway from the peripheral nerve endings is based on the evidence that some CoVs appear to be capable of penetrating the CNS through the cribriform plate of the ethmoid bone, even if the olfactory bulb is efficient enough to control viral invasion [113]. According to Li et al. [113], mechanoreceptors and chemoreceptors in the lung and respiratory tract can act as a possible retrograde pathway for SARS-CoV-2, as the nucleus of the solitary tract receives sensory information from these anatomical structures. Indeed, a dysfunction of the cardiac-respiratory control centers in the medulla oblongata would aggravate the symptoms till death [113].

However, the neurogenic hypothesis of respiratory failure is not supported by other researchers [114], as they argue that patients with COVID-19 pneumonia do develop hypoxia and low CO_2_ levels accompanied by increased respiratory rate. While these patients can breathe spontaneously, they do it with great effort; thus, a respiratory failure resulting from a neurological origin would be characterized by a reduced respiratory rate, low oxygen levels, and high CO_2_ levels [114]. Further virologic, histopathological, and immunohistochemical studies are necessary to demonstrate a specific neurotropism of SARS-CoV-2 for the brain respiratory control centers.

The hypotheses behind SARS-CoV-2 transsynaptic propagation are further corroborated by other studies demonstrating that the virus may use a transsynaptic route for infecting the CNS. One of the first pieces of evidence was provided in 1986 by Gosztonyi [115], who described the axonal transport of viral nucleic acid of some neurotropic viruses. In particular, he described that the rabies virus (RV) could be transmitted to other neurons by transsynaptic passage, without involving the complete virus replication, thus reaching various brain areas. Li et al. [105] demonstrated that HEV was able to propagate into CNS via transsynaptic routes. Namely, the peripheral inoculation of HEV in both piglets and rodents results in encephalomyelitis via the primary motor cortex, where membranous-coating-mediated endo-/exocytosis events favor the HEV transsynaptic transfer. In a recent review, Taylor and Enquist [116] deeply described the axonal route of propagation of several neuroinvasive viruses, including herpes simplex, varicella-zoster, pseudorabies, *Rhabdoviridae* (including RV), *Flaviviridae*, vesicular stomatitis (VSV), and Theiler’s murine encephalitis virus (belonging to the *Picornaviridae*). They also described the mechanisms by which viruses were able to move in and out axons, both anterogradely or retrogradely, thanks to coupled or separate transports (mediated by vesicles or not, respectively) [116].

The anterograde and retrograde transsynaptic propagation lead researchers to adopt this viral feature for mapping the axon transports of neuronal impulses, viruses, and other factors. In this context, the transsynaptic transport of VSV has been used for tracing and mapping neuronal circuits by using specific virus-labeling techniques [117,118]. Of note, transsynaptic propagation is not a prerogative of CNS viruses. For instance, the measles virus (MV) can reach the CNS and cause subacute sclerosing panencephalitis, which is often fatal. The CNS complications of MV infection are known to be the results of transsynaptic viral propagation thanks to the binding between MV envelope F protein and several host proteins, including hemagglutinin and neurokinin-1 [119].

Taken together, these observations support the concept that some viruses, including respiratory viruses like the SARS-CoV-2, may propagate through the CNS via a transsynaptic transport.

### 5.3. The Role of Angiotensin II Converting Enzyme Receptor

The cell invasion of SARS-CoV-2 and its rapid replication seem to be supported by the ACE2 receptor [120]. The damaging effects of angiotensin II may be enhanced because of the depletion of the ACE2 receptor on the cell membrane, which leads to an acute deterioration in lung function. Therefore, the down-regulation of the ACE2 receptor could put the hypertensive and diabetic population at higher risk for COVID-19 due to the increase in angiotensin II. A hypothesis related to this issue is that ACE inhibitors, when used in patients with COVID-19, can lead to an increased expression of ACE2, thus probably making the cells more vulnerable to SARS-CoV-2 infection [120]. A study examining the risk factors for mortality in patients with COVID-19 found that 40% of the deceased people presented single or multiple comorbidities, with high blood pressure being the most common (30%) [121].

The neurovirulence of SARS-CoV-2 could be related to the degree of expression of the ACE receptor in the CNS, although this receptor is expressed in endothelial cells, so it is necessary to further investigate its role in the etiopathogenesis of some neurological complications, such as stroke [23]. The viral S protein might allow the virus interaction in brain microcirculation with ACE2 receptors expressed in the capillary endothelium, possibly leading to endothelial cells infection and subsequent spreading to the neurons after that the endothelial damage has occurred [24].

### 5.4. Hematogenous Propagation and the Role of the Blood-Brain Barrier

Damage of the epithelial barrier by CoVs may occur, thus allowing the virus to reach the bloodstream or the lymphatic system and to spread to other tissues, including the brain. In this scenario, however, it is important to distinguish between the nasal olfactory epithelium and the nasal respiratory epithelium. While the former has been indicated as the main route for the trans-synaptic propagation of CoVs, the latter seems to be involved in the hematogenous propagation [78]. Nevertheless, it is not well clearly understood how this could take place, although the BBB seems to be involved.

Two hypotheses have been proposed for the crossing of the BBB by SARS-CoV-2. The first mechanisms would involve the infection and transport across vascular endothelial cells, which express ACE2 and, as such, are at risk for SARS-CoV-2 infection [78]. SARS-CoV-2 particles have been found in capillary endothelia and neurons of a frontal lobe specimen from an autopsy case study [122]. In particular, viral particles were packaged in intraneuronal dilated vesicles, and endocytosis or exocytosis of viral particles across endothelial cells were detected by electron microscopic imaging [78]. As soon as the virus enters vascular and neuronal cells, it might interact with ACE2 on neurons, glia, and vessels, and then begin a cycle of viral budding, thus further damaging both vascular and neuronal tissue [24].

The second hypothesis is based on the so-called “Trojan horse mechanism,” through the infection of leukocytes that pass the BBB [35]. As lymphocytes, granulocytes, and monocytes all express ACE2, the SARS-CoV might able to infect them [49,123,124,125], and it is likely that SARS-CoV-2 too may act in the same manner. Moreover, the COVID-19-related systemic inflammation would increase the BBB permeability, thus facilitating the invasion of the CNS by the infected immune cells [126].

### 5.5. Other Mechanisms

COVID-19-related hypoxia may be responsible for indirect neuronal damage as it induces anaerobic metabolism in the CNS cells, ischemia, interstitial edema, and vasodilatation in the cerebral circulation, which eventually causes stroke, syncope, and anoxic crisis [127].

The fact that CoVs can infect macrophages, astroglia, and microglia makes it possible for the host’s immune-mediated response to playing a role. In some patients who died because of COVID-19, a multiple organ failure and a hyperinflammatory syndrome (the “cytokine storm”) were hypothesized as possible underlying causes [81]. In this context, a previous study in mice showed T-lymphocyte infiltration into the CNS and significantly increased levels of the proinflammatory cytokine IL-6 and the chemokine monocyte chemoattractant protein-1 after CoV exposure [128].

Finally, in genetically predisposed individuals, the persistence of CoVs in some CNS resident cells cannot be excluded, where they would act as a cofactor of clinical exacerbations. Serological techniques have identified CoVs in various neurological diseases, such as Parkinson’s disease, MS, and optic neuritis [129,130,131,132]. Therefore, it was proposed that a persistent CoV infection might be a pathogenic factor in the development and course of some neurological diseases. For instance, infectious agents may play a triggering role in MS, with viruses being the most likely culprit in genetically predisposed individuals [133].

Taken together, all the mechanisms discussed here might, at least in part, explain why and how SARS-CoV-2 could be moved in or within the CNS despite the low expression of ACE2 receptor in the brain. Further studies are needed to better understand these pivotal aspects of neuroinfection.

## 6. Unmet Needs and Conclusive Remarks

The research on the neurological manifestations of COVID-19 has recently made significant progress, although the exact neuropathogenic mechanisms of SARS-CoV-2 are not yet completely clear. Essential questions are emerging with the identification of people with COVID-19 and CNS involvement. Although these patients may exhibit a wide range of neurological complications, it remains to be determined whether these can be an indirect and unspecific consequence of the pulmonary disease, hypoxia, or generalized inflammatory state on the CNS, or if they may rather reflect a direct viral-related neuronal damage. Some symptoms, such as headache and unsteadiness, are non-specific manifestations of several viral infections but, in some cases, they might accompany more severe diseases, such as meningitis, encephalitis, and stroke.

A hematogenous versus a transsynaptic propagation is still debated, as well as the role of the ACE2 receptor, the impact of hyperimmune response, and the viral persistence within some CNS cells. The different levels and severity of human neurotropism and neurovirulence in patients with COVID-19 might be explained by a combination of viral and host factors and their interaction. Although researchers are yet to elucidate the real degree of neurovirulence of SARS-CoV-2, there is a demonstration of its presence in the CSF or tissue samples at autopsy. However, in the current epidemic, some difficulties may occur in performing MRI or a lumbar puncture, especially in severely affected patients and in those admitted in intensive care units. Nevertheless, it remains of pivotal importance for all patients with altered consciousness or any unexplained neurological manifestation to receive an accurate neurological exam and appropriate instrumental investigations (i.e., neuroimaging, EEG, evoked potentials, CSF), when necessary [134].

Another warning related to this topic is that lymphopenia in immunosuppressed patients with COVID-19 can be a serious risk factor; these are not only patients with cancer or with systemic autoimmune diseases but also patients with neurological disorders. Indeed, taking high-dose corticosteroids or immunosuppressive/biological treatments is relevant for diseases like cerebral vasculitis, neuromyelitis optica, neurosarcoidosis, polymyositis, myasthenia gravis, or MS, and the scientific community should rapidly develop ad hoc guidelines for the management (especially in terms of reevaluation of dosages and treatment cycles) of these diseases during the COVID-19 era.

Another relevant consideration concerns the possibility of developing anti-COVID-19 drugs to cross the BBB and to selectively target the SARS-CoV-2 inside the brain. The role of the BBB needs to be further explored in patients with COVID-19. Hence, the possible neuroinvasion may be a significant mechanism to take into account for treating and preventing COVID-19. In this context, the design of safe and effective brain penetrating drugs would be very helpful in preventing and possibly treating the neurological complications of COVID-19, although the studies on this “cutting-edge topic” are still at their beginning [135].

The differences in the sequence of spike proteins between SARS-CoV and SARS-CoV-2 will enable scientists to identify epitopes in COVID-19 patients for the development of monoclonal antibodies against this virus. Basic research studies on SARS-CoV-2 and host interactions are the key to several unanswered questions in the prevention and control of the disease, including the challenging question of why not all patients with COVID-19 show neuroinvasion and why, among those experiencing neuroinvasion, not all show neurotropism or neurovirulence [136]. Moreover, the difference in terms of neurological involvement and pathomechanisms between the current pandemic and the SARS and MERS infections needs to be further studied.

Lastly, longitudinal neurological assessments of patients after their recovery will be crucial in the understanding of the natural history of COVID-19 in the CNS and for monitoring potential neurological sequelae. Reaching a more global vision of COVID-19 neuroinfection is also crucial, for instance how SARS-CoV-2 may affect the clinical expression of other infections or co-infectious diseases within the CNS (i.e., the human immunodeficiency virus).

Further studies on clinical features of patients and pathogenetic mechanisms will provide guidance to deal with this pandemic infection, which seems to go well beyond pneumonia and whose multifaceted aspects warrant an urgent need for multidisciplinary and multidimensional research.

## Abbreviations

ACE2	angiotensin-converting enzyme-2
BBB	blood-brain barrier
CNS	central nervous system
CoV	coronavirus
CoVs	coronaviruses
CQ	chloroquine
CSF	cerebrospinal fluid
CT	computed tomography
DPP4	dipeptidyl peptidase-4
EEG	electroencephalography
GBS	Guillain-Barré syndrome
HCQ	hydroxychloroquine
HEV	swine hemagglutinating encephalomyelitis virus
IL	interleukin
MERS	Middle East Respiratory Syndrome
MRI	magnetic resonance imaging
MS	Multiple Sclerosis
MV	measles virus
ORNs	olfactory receptor neurons
RCTs	randomized clinical trials
RSV	respiratory syncytial virus
RT-PCR	real-time reverse polymerase-transcriptase chain reaction
RV	rabies virus
SARS	Severe Acute Respiratory Syndrome
SUSs	sustentacular cells
TMPRSS2	transmembrane protease serine 2
TNFα	tumor necrosis factor α
VSV	vesicular stomatitis virus
WHO	World Health Organization
